# Towards Effective Treatment of Glioblastoma: The Role of Combination Therapies and the Potential of Phytotherapy and Micotherapy

**DOI:** 10.3390/cimb46120859

**Published:** 2024-12-19

**Authors:** Ludovica Gaiaschi, Maria Grazia Bottone, Fabrizio De Luca

**Affiliations:** Laboratory of Cell Biology and Neurobiology, Department of Biology and Biotechnology “L. Spallanzani”, University of Pavia, Via Ferrata 9, 27100 Pavia, Italy; ludovica.gaiaschi@unipv.it (L.G.); mariagrazia.bottone@unipv.it (M.G.B.)

**Keywords:** glioblastoma, resistance, conventional therapy, innovative therapy, combined therapy, natural adjuvant

## Abstract

Glioblastoma multiforme (GBM) is one of the most aggressive and difficult-to-treat brain tumors, with a poor prognosis due to its high resistance to conventional therapies. Current treatment options, including surgical resection, radiotherapy, and chemotherapy, have limited effectiveness in improving long-term survival. Despite the emergence of new therapies, monotherapy approaches have not shown significant improvements, highlighting the need for innovative therapeutic strategies. Combination therapies appear to be the most promising solution, as they target multiple molecular pathways involved in GBM progression. One area of growing interest is the incorporation of phytotherapy and micotherapy as complementary treatments, which offer potential benefits due to their anti-tumor, anti-inflammatory, and immunomodulatory properties. This review examines the current challenges in GBM treatment, discusses the potential of combination therapies, and highlights the promising role of phytotherapy and micotherapy as integrative therapeutic options for GBM management.

## 1. Challenges in Glioblastoma Treatment

Glioblastoma (GBM) is the most aggressive and common primary brain tumor in adults, marked by rapid growth, extensive invasion into surrounding tissue, and resistance to therapies. The World Health Organization (WHO) redefined GBM in 2021 as an isocitrate dehydrogenase (IDH) wild-type diffuse astrocytic glioma. Diagnosis is confirmed by evidence of microvascular proliferation, necrosis, telomerase reverse transcriptase promoter mutations, EGFR (epidermal growth factor receptor) gene amplification, or chromosome copy number alterations (+7/−10) [1].

A significant challenge in treating GBM is the blood–brain barrier (BBB), which restricts most drugs from entering the central nervous system (CNS), limiting the efficacy of systemic chemotherapies and hindering the development of new treatments [2]. Additionally, the tumor microenvironment (TME) is hostile, featuring hypoxic regions that promote an aggressive phenotype, enhance tumor invasion, and stimulate angiogenesis. This, along with the immunosuppressive nature of the TME, creates a protective niche that renders GBM particularly refractory to treatment [3,4].

The heterogeneity of glioblastoma, both within individual tumors and among different patients, presents significant challenges for developing effective therapeutic strategies. GBM tumors are composed of diverse populations of cancer cells, each with unique genetic and epigenetic characteristics, which makes it difficult to effectively target all cells with a single approach [5,6]. This diversity also includes cancer stem cells, which have the ability to self-renew and often drive tumor regrowth after treatment, further complicating efforts to achieve long-lasting therapeutic success [7]. Moreover, different tumor regions exhibit varying levels of proliferation, hypoxia, and invasiveness, further complicating therapy [8]. Moreover, GBM cells harbor numerous mutations, affecting key pathways such as EGFR, PTEN, and TP53, leading to multiple resistance mechanisms [9,10]. Consequently, treatments like chemotherapy and radiation often fail to eradicate the tumor completely, emphasizing the need for personalized, multi-targeted therapeutic approaches to address this complexity.

## 2. The Inadequacy of Temozolomide Treatment

Chemotherapy is pivotal in the standard care of glioblastoma, with temozolomide (TMZ), an oral alkylating agent, remaining the cornerstone. TMZ is administered alongside radiotherapy after surgical resection, as in the Stupp protocol [11]. Its ability to cross the blood–brain barrier (BBB) and synergize with radiation prolongs survival; however, resistance frequently develops during treatment [12].

Elevated expression of O6-methylguanine-DNA methyltransferase (MGMT) significantly enhances resistance to TMZ. Proteomic and metabolomic analyses have revealed a strong link between an unmethylated MGMT promoter and the activation of DNA damage repair (DDR) pathways [13]. The role of DDR has been validated in both MGMT-deficient GBM cells [14], and through in vivo and ex vivo studies [15,16]. Additionally, the epidermal growth factor receptor variant III (EGFRvIII) has been shown to activate the NF-κB pathway, crucial in DDR processes. Proteins like E2F1 and RAD51AP1 also play key roles in the DDR mechanisms of EGFRvIII-positive GBM cells [17,18,19].

Epigenetic modifications, such as H3K9ac, have been found to upregulate MGMT expression, further contributing to TMZ resistance [20]. The MGMT status also correlates with differential immune responses, suggesting its potential as a predictor of treatment outcomes [21]. Other epigenetic changes, such as histone modifications [20,22] and the role of non-coding RNAs, have been linked to resistance mechanisms. For example, downregulated miR-34a or high levels of miR-1246 have been associated with increased TMZ resistance [23,24] through their interaction with tumor suppressor genes. Additionally, long non-coding RNAs (lncRNAs), such as the lncRNA HOXD-AS2/STAT3 feedback loop [25], and the recently discovered LINC00470/EGR2/SOX4 axis [26], have shed new light on resistance modulation.

Further resistance mechanisms, including metabolic adaptations and nutrient availability, especially under hypoxia, are receiving attention [27,28]. Hypoxia-inducible factors and oxidative phosphorylation [29] have been shown to promote cell survival, while autophagy helps counteract TMZ-induced cytotoxicity [30]. Other significant players include ABC transporters, efflux pumps, and transcription factors like the EGR protein family, as well as proteins such as metalloproteinases and annexins, all of which contribute to poor TMZ responses [31,32,33].

Recent discoveries about TMZ-induced hypermutation have revealed insights into tumor recurrence, chemoresistance, and its impact on prognostication and clinical trial design [34]. Meanwhile, the safety profile of TMZ continues to be debated, with studies presenting conflicting data on its association with neurocognitive disorders [35,36,37,38] and recognizing risks of secondary neoplasms and myelosuppression, which may hinder immune surveillance and promote tumor progression [39,40,41].

## 3. The Limitations of Monotherapy

Before the advent of TMZ, nimustine (ACNU), carmustine (BCNU) and lomustine (CCNU) were long used for the treatment of gliomas [42]. They are nitrosourea compounds that act through the alkylation (DNA cross-linking) and carbonylation of proteins. Unfortunately, a high frequency of toxicity profiles was reported. In recent years, these chemotherapy drugs have been rediscovered. The possible efficacy of ACNU against TMZ-resistant brain tumor cells has been highlighted in preliminary in vitro and in vivo studies [43], and it also seems to be an excellent candidate for convection-enhanced delivery (CED) [44]. Studies have also been reported to evaluate BCNU’s actual positive effect in clinical use on brain tumors with a high grading. The in vivo results are encouraging but do not show superior efficacy compared to that observed with TMZ [45,46,47]. Despite this, research for new delivery routes that guarantee greater availability at the site of need is ongoing using in vitro and ex vivo models [48,49]. Similarly, despite promising in vitro results [43], no clinically relevant improvement appears to be given by the use of CCNU [50,51]; on the contrary, severe side effects are reported [52]. However, CCNU is administered in combination with procarbazine, a methylating agent, and vincristine, which inhibits microtubule formation, for recurrent GBM or patients who do not respond to TMZ, according to what is called the PVC regimen [53].

Other chemotherapy drugs have been considered for the treatment of GBM. Alkylating agents such as cisplatin or carboplatin have been evaluated. Cisplatin is a highly effective chemotherapeutic agent capable of targeting actively and inactively duplicating cells; however, its application in treating GBM is restricted due to significant systemic toxicity and poor penetration into brain tumor tissue. In this regard, previous studies showed how direct delivery of this chemotherapeutic agent to the brain could improve patient outcomes [54,55]. Similar results were also obtained, through clinical studies, after carboplatin administration [56,57]. Despite this, the use of fourth-generation platinum compounds, capable of forming platinum-DNA adducts and mainly intrastrand cross-links, remains a promising resource [58].

Research into the immune microenvironment of glioblastoma has sparked significant interest in testing immunotherapies. Chimeric antigen receptor (CAR) T cells and CAR natural killer (CAR-NK) cells are cutting-edge immunotherapies. CAR T-cell therapy involves genetically engineering patients’ T cells to express receptors to antigens on the surface of GBM cells; CAR-NK cell therapy uses natural killer cells, either from the patient or a donor, which are also engineered to express CARs targeting GBM cells. NK cells have innate tumor-killing abilities and less toxicity compared to CAR-T cells [59,60]. In vitro and preclinical studies have shown promising results for cell therapies [61,62,63,64], but these findings have not been entirely satisfactory partially due to the immunosuppressive tumor microenvironment: thus, new GBM-targeting CAR-T cells countering TGF-β-mediated immune suppression in the TME are being developed on murine models [65]. However, despite this enthusiasm, clinical trials involving immunotherapy in glioblastoma have so far not demonstrated a clear survival benefit for patients. A retrospective study of adult patients diagnosed with first-recurrence GBM did not show extended overall survival resulting from the administration of Pembrolizumab [66], an immune checkpoint inhibitor blocking programmed death receptor-1 (PD-1); consistently, no positive results emerged in patients with recurrent high-grade gliomas or glioblastoma [67,68]. Nivolumab, also a PD-1 inhibitor, gave more promising results in a GBM-bearing rodent model [69] and in GBM patients [70,71]; in particular, systemic immune responses seemed to be enhanced by nivolumab. Despite this, the treatment’s effectiveness was hindered by the tumor’s anti-inflammatory mechanisms, reducing the overall clinical impact [72]. New hope is represented by IGV-001, a personalized approach where patient’s tumor cells are treated with an agent to induce immunogenic cell death, encapsulated in small devices that are then implanted into the patient to trigger a strong immune response. The therapy gave good results in GL261-bearing mice [73] and is currently in clinical trials for newly diagnosed GBM patients [74,75].

Moreover, recent research in cancer vaccines for glioblastoma highlights a variety of innovative strategies. Personalized mRNA [76,77,78,79] or DNA vaccines [80,81,82,83], designed and evaluated through in silico and omics approaches [76,77,78] and tested in preclinical models [79,80,81,82,83], seemed very promising as tools for immunotherapy; however, it has also been noticed that they contribute to the immunosuppressive environment within the tumor, helping the GBM evade the body’s immune system [83], which could limit the success of immunotherapies or other treatments. Similarly, dendritic cell vaccines, promising in the preliminary studies, did not achieve a mean overall survival improvement in clinical studies [84,85,86,87,88]; on the contrary, treatment-emergent adverse effects on the central nervous system were noticed in a phase II study, where patients showed mostly mild adverse effects (injection-site reactions, flu-like symptoms, and bone pain) and more than half experienced serious seizures, falls, or cerebral edema [88].

Great space and interest are being given to oncolytic virus (oV) therapy, particularly that mediated by herpes simplex virus [89,90,91], flavivirus, and adenovirus [92,93], in preclinical settings [89,90,91,93] and in in vitro studies [92]. Oncolytic virus therapy in recent years has shown great efficacy in in vitro and in vivo GBM models, demonstrating substantial antitumor activity and favorable tolerance [94], but it is also true that oV sensitivity varies from patient to patient [95] and that its efficacy could be limited by insufficient delivery to tumors after systemic injection and the propensity of oVs to induce the expression of immune checkpoints. For this reason, research groups are working to improve the performance of this therapeutic strategy, targeting genes encoding immune checkpoint proteins, e.g., PD1 [96,97], or suppressing IL-2 [97,98] in mouse cancer models. Despite these advances, immunotherapy in glioblastoma remains largely ineffective as a single therapy. To date, targeted therapies like nivolumab [72,99,100,101] and pembrolizumab [66,67,102,103] (PD-1 inhibitors) alone have also shown limited success in GBM trials.

Nevertheless, aiming to impair specific molecules or pathways that drive GBM growth and resistance to standard treatments with targeted therapies seems to be a promising new frontier. Due to the known overexpression or mutation of receptor tyrosine kinases (RTKs) in GBM, RTK inhibitors have been developed. For example, drugs targeting EGFR (epidermal growth factor receptor), especially the EGFRvIII variant, such as afatinib, which gave interesting in vitro and in silico results [104,105], dacomitinib, studied both in vitro and in mice models [106], and erlotinib, whose possible therapeutic interest has been validated in silico, have shown limited success [107]—likely due to tumor heterogeneity and resistance mechanisms—or have not yet moved to more advanced stage studies. Drugs like imatinib [108,109,110] are being investigated in vitro, in murine models [111], and in clinical settings [112,113] for their potential role in targeting the platelet-derived growth factor receptor (PDGFR). However, the results have been mixed. Studies have identified both resistance mechanisms and significant variability in cellular responses, highlighting the challenges in achieving consistent therapeutic outcomes with this approach. Anti-angiogenic therapies like bevacizumab, a VEGF (vascular endothelial growth factor receptor) inhibitor, are FDA-approved, but their impact on overall survival remains modest, likely due to compensatory pathways [114] and the presence of different molecular subtypes of GBM [115]. Thus, their use as a monotherapy did not give any benefit in terms of overall survival and quality of life (QoL) improvements in both clinical and preclinical studies [116,117,118,119,120].

A similar rationale has guided research interest in glioblastoma therapy towards PI3K/AKT/mTOR (phosphoinositide 3-kinase/ protein kinase B/ mammalian target of rapamycin) pathway inhibitors, which should impend cell survival and growth and regulate protein synthesis and cell metabolism. Drugs like buparlisib (PI3K inhibitor) [121,122,123,124,125], everolimus (mTOR inhibitor) [126,127,128,129,130], and ipatasertib (AKT inhibitor) [131,132,133] have been tested in vitro and in vivo (buparlisib and everolimus reached clinical studies), but their success seemed limited by compensatory mechanisms and toxicity concerns. In the context of cellular energy homeostasis, autophagy and proteasomes have gained attention, while marizomib, a proteasome inhibitor, has been studied for its possible beneficial effect in cancer treatment. However, the latter did not demonstrate any meaningful benefit and also seemed to exacerbate other adverse effects of chemotherapy [134,135,136].

Inhibitors targeting enzymes like isocitrate dehydrogenase (IDH) (in mutant GBM), LDH (lactate dehydrogenase), and GLS (glutaminase) are being studied for their potential to starve GBM cells. Ivosidenib, an IDH1 inhibitor whose effects were evaluated in silico [137], showed promise in IDH-mutant GBMs by targeting the metabolic vulnerabilities of these tumors [138]. LDH has been recognized as a prognostic marker of invasiveness in preliminary studies and its inhibition seemed to be favorable for the outcome. Nevertheless, inconsistent results have been recorded in preclinical studies, showing the heterogeneous response from molecularly different GBM cells and the activation of compensatory metabolic pathways [139,140,141]. Also, GLS inhibition evaluation gave promising preclinical results when used in combined therapy [142,143,144,145,146,147].

Since GBM cells are metabolically very active, in the context of metabolic targeting, different forms of starvation and their effect on tumor growth have been considered; in addition to the effect of glutamate homeostasis mentioned above, the ketogenic diet with glucose starvation, arginine deprivation, and the role of iron in tumor growth have also been investigated. The maintenance of low glucose levels has given positive results not only in combined therapy in preclinical and clinical studies [121,148,149] but also in a case report of monotherapy [150]. Despite this, some points are poorly clarified; in fact, the ketogenic diet in murine models seems to promote an immunosuppressive phenotype in macrophages, thus limiting the clinical relevance of the findings [151].

In addition, the importance of targeting DNA damage repair and chromatin organization mechanisms in GBM has gained attention as it offers the opportunity to modulate the epigenetic regulation of gene expression and chromatin organization as well as DDR (DNA damage repair) pathways. Histone deacetylase (HDAC) inhibitors such as panobinostat [152,153,154,155,156,157] have been tested in GBM in vitro and in vivo using the multiomics approach, showing some efficacy—especially when combined with other treatments. Valproic acid, an antiepileptic drug used for its HDACi activity, has been repurposed for GBM treatment due to its promising effects in vitro [158,159]; however, the efficacy of monotherapy in clinical settings was also scarce in this case [160]. Similarly, poly(ADP-ribose) polymerase (PARP) inhibitors targeting DNA repair mechanisms, like niraparib [161,162,163] and olaparib [162,164,165,166,167,168,169], have shown potential positive effects in preclinical trials; however, even in this case, the most encouraging results in terms of possible benefits from introduction into clinical practice have emerged from their use in combination therapy.

The limited success rate of several investigations on new drugs and the urgency to identify valid glioblastoma treatments lead to a drug repurposing approach, which is cost-effective and needs less time to bring FDA-approved drugs to clinical trials. Metformin, used to manage type 2 diabetes, is known to reduce gluconeogenesis, enhance peripheral glucose uptake, and increase metabolism. Due to its effects on metabolic pathways and cellular signaling, metformin gained attention as an anti-GBM treatment both in in vitro and in clinical studies [170,171,172,173]. For its involvement in metabolism, disulfiram, an aldehyde dehydrogenase inhibitor used in alcoholism management, has been tested on GBM, revealing promising adjuvant efficacy in vitro [174,175], as also confirmed by in vivo and clinical studies [175,176]. Chloroquine’s and hydroxychloroquine’s ability to modulate autophagy and cell metabolism made these drugs, used to prevent and treat malaria, of interest for their use against GBM in vitro [157,177]; however, once again, they alone did not reach clinically relevant results [178,179].

Not only is chemotherapy being investigated for its potential role in treating deadly cancers, but innovative therapeutic approaches are also being extensively studied and developed. Proton therapy, sonodynamic or photodynamic therapy, hyperthermia, and tumor treating fields have been recognized as non-invasive techniques that could give beneficial results for GBM patients’ QoL thanks to their limited adverse systemic effects. Proton therapy, with its unique Bragg peak effect, offers the advantage of precise tumor targeting, making it a promising approach in cancer treatment. However, while it shows significant potential, its long-term effectiveness compared to conventional therapies remains under ongoing evaluation. In combination therapies, proton therapy has demonstrated beneficial adjuvant effects, as noted in previous clinical studies [180]. On the other hand, the results are less clear when proton therapy is used as a monotherapy in clinical trials or in rat models, with more research needed to fully understand its independent efficacy. [181,182]. Sonodynamic [183,184,185,186] and photodynamic [187,188,189,190] therapies, extensively studied across various settings, use energy waves to activate a photosensitizer or sonosensitizer within the tumor, leading to reactive oxygen species (ROS) production. While promising, their efficacy as monotherapies for aggressive tumors like glioblastoma is still unclear due to concerns about penetration depth and reliance on oxygen [187,191]. Hyperthermia therapy consists of heating the tumor to 40–45 °C to stimulate immune response and make cells more sensitive to chemotherapy, thus making hyperthermia inefficient as a monotherapy [191,192,193]. Moreover, the complex vascular network seems to contribute to inconsistent responses to this treatment, as shown in in silico approaches [194]. Most of all, in recent years, tumor treating fields (TTFs) have gained credibility. TTF has been approved as adjuvant therapy for glioblastoma, and its efficacy as a monotherapy has been proved in clinical studies [195]. It uses alternating electric fields to disrupt cancer cell division, and has shown promise as a novel CNS drug delivery strategy by inducing transient BBB permeabilization in in vitro and in vivo models [196,197]. Although its safety profile as a monotherapy has been demonstrated, with fewer side effects than the Stupp protocol and other possible chemotherapies [198], many aspects are still unclear in relation to the variability of responses between different patient samples [199]. Furthermore, the greatest success of this therapy in GBM patients was once again recorded when used in conjunction with other treatments [200]; unfortunately, as a monotherapy, it is only successful when used continuously for more than 18 h a day, which raises important compliance issues [201]. In this context, a recently concluded phase I study assessed the safety of a portable device for tumor treating fields therapy, NovoTTF-200A (NCT03477110). A phase II study on Optune^®^ System (NCT04492163) also gave promising results.

## 4. Beyond Monotherapy: The Power of Combined Treatments

Monotherapy has shown limited success due to the complexity and adaptability of GBM cells, combining therapeutic approaches and leveraging different mechanisms of action to overcome tumor heterogeneity, improve treatment efficacy, and delay or prevent resistance by targeting multiple pathways simultaneously. Therefore, numerous studies are underway to evaluate the safety and efficacy of different combination therapies in the treatment of GBM [202] (Table 1).

In early phase I, the safety of a new dual-action alkylating agent, tinostamustine, which appears capable of targeting both cancer cells and the tumor microenvironment, is under analysis as an adjuvant in patients who completed concomitant treatment with temozolomide and radiation. Patients are also being recruited for studies whose objective is to evaluate the safety of blockers of DNA damage repair mechanisms, such as AZD1390, niraparib, pamiparib, and olaparib, in combination with standard-of-care fractionated radiotherapy. Phase I/II studies have also evaluated the effect of pamiparib (or BGB-290) in combination with TMZ (NCT03914742), but the results have not been published yet. Additionally, various combinations of targeted therapies are being explored in clinical trials to enhance therapeutic efficacy and overcome current treatment limitations. For instance, LY3214996, an ERK1/2 inhibitor, is being tested in combination with abemaciclib, a CDK4/6 inhibitor. Another promising combination includes AB154, an anti-TIGIT (T cell immunoreceptor with Ig and ITIM domains) immune checkpoint inhibitor, alongside AB122, an anti-PD-1 known as zimberelimab. Additionally, defactinib, a focal adhesion kinase inhibitor, is being studied in combination with VS-6766, a RAF/MEK inhibitor. In addition, in light of the importance of personalized therapy, a study has been opened for the evaluation of the effects, in combination with standard-of-care treatments, of a cocktail of up to 3 FDA-approved drugs from a panel of compounds selected through high-throughput screening of cancer stem cells derived from the patient’s tumor [202].

**Figure 1:**
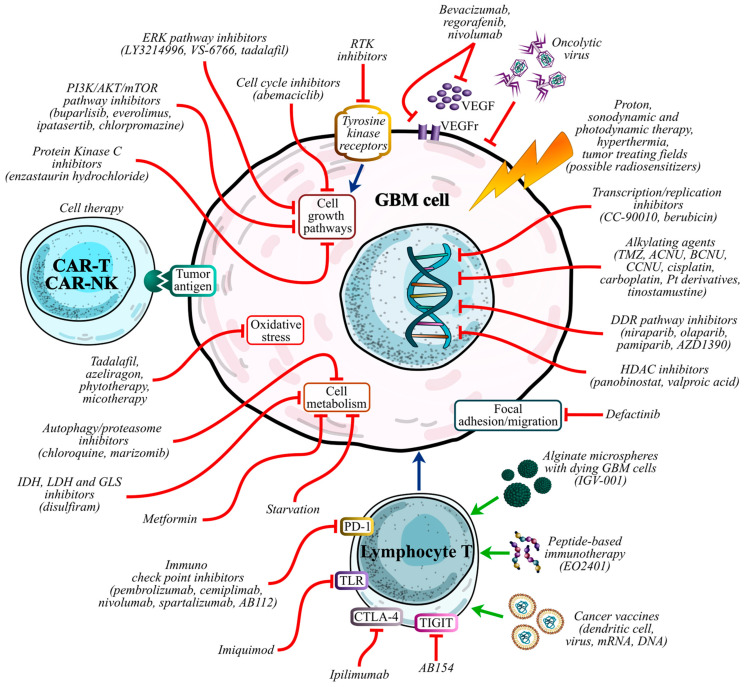
Schematic representation of therapeutic strategies for glioblastoma. The diagram illustrates key molecular targets and pathways involved in GBM progression, highlighting therapies discussed in this review. Specifically, alginate microspheres containing dying GBM cells, peptide-based immunotherapy, and cancer vaccines act as positive regulators of T lymphocyte activity. In parallel, immune checkpoint inhibitors and various agents such as imiquimod, ipilimumab, and AB154 exert inhibitory effects on specific receptors of these leukocytes. On the other hand, cell-based therapies using CAR-T and CAR-NK cells mediate an inhibitory effect on glioblastoma cells through recognition and interaction with specific tumor antigens. Transcription/replication inhibitors, alkylating agents, DDR pathway and HDAC inhibitors directly target tumor cells at the nuclear level, regulating transcription, replication, and gene expression. Defactinib inhibits mechanisms of focal adhesion and cellular migration. Specific molecules, e.g., autophagy/proteasome, IDH, LDH, and GLS inhibitors, metformin, and starvation therapies, block the metabolic processes of neoplastic cells. Tadalafil, azeliragon, phytotherapy, and micotherapy act as modulators of intracellular oxidative stress levels. Cell growth pathways are inhibited by protein kinase C, PI3K/AKT/mTOR pathway, ERK pathway, cell cycle, and RTK inhibitors. In particular, RTK inhibitors block cell growth pathways by interacting with specific tyrosine kinase receptors. Bevacizumab, regorafenib, and nivolumab inhibit both VEGF molecules and its receptors. Additionally, oncolytic viruses mediate an inhibitory effect on GBM cells, as do proton therapy, sonodynamic and photodynamic therapy, hyperthermia, and tumor treating fields. Red arrows indicate inhibitory effects, while green arrows represent activation or promotion of therapeutic pathways. Abbreviations: ACNU (nimustine), BCNU (carmustine), CAR-NK (chimeric antigen receptor natural killer cell), CAR-T (chimeric antigen receptor T-cell), CCNU (lomustine), DDR (DNA damage repair), GBM (glioblastoma), GLS (glutaminase), HDAC (histone deacetylase), IDH (isocitrate dehydrogenase), LDH (lactate dehydrogenase), PI3K/AKT/mTOR (phosphoinositide 3-kinase/protein kinase B/mammalian target of rapamycin), RTK (receptor tyrosine kinases), TIGIT (T cell immunoreceptor with IG and ITIM domains), TMZ (temozolomide), VEGF (vascular endothelial growth factor), VEGFr (vascular endothelial growth factor receptor).

In phase I, different drugs are being tested in combination with standard-of-care radiation therapy and temozolomide: for example, chlorpromazine or cannabinoids, known for their antipsychotic, anti-inflammatory, and anti-angiogenesis properties; chloroquine, which is used as antimalaric; or specific inhibitors like tadalafil, hosphodiesterase type 5 (PDE5) inhibitors, or CC-90010, bromodomain and extra-terminal motif (BET) inhibitor. In addition, new types of therapy are under consideration in phase I/II studies, such as the association of AGuIX nanoparticles, a radiosensitizer, personalized dendritic-cell vaccines, and radiotherapy in combination with TMZ. There is growing hope in the potential of immunomodulatory drugs to improve cancer treatment outcomes. PD-1 inhibitors, including nivolumab, pembrolizumab, cemiplimab, and spartalizumab, are currently being tested in combination with other immune-targeting therapies. For example, they are paired with CTLA-4 inhibitors like ipilimumab, which is also being studied alongside radiotherapy in an active phase III trial. Other combinations under investigation include PD-1 inhibitors with dual ILT2/ILT4 antagonists, such as NGM707, TIGIT inhibitors like ASP8374, and TIM-3 inhibitors, such as MBG453. It should be noted that a phase IV study on pembrolizumab in combination with standard therapy is currently open. A phase II study using regorafenib, a VEGFR inhibitor, taken together with nivolumab (NCT04704154), has recently been completed, but the results of this study do not support further evaluation of regorafenib combined with nivolumab in GBM [202,203]. Immunomodulators and cancer therapeutic vaccines, such as nivolumab and bevacizumab with EO2401 or imiquimod—an activator of toll-like receptor 7—with the GBM6-AD vaccine, are raising interest. In 2024, the phase II studies of pembrolizumab, another PD-1 inhibitor, in combination with lerapolturev (NCT04479241) or SurVaxM (NCT04013672), an oncolytic virus and a peptide vaccine targeting survivin, have been completed; however, neither progression-free survival nor overall survival were reported at the time of the review. In a phase II study, azeliragon, an anti-inflammatory drug, is currently being tested in combination with radiation therapy to explore its potential to enhance treatment outcomes. Other innovative approaches under investigation include UCPVax, an anti-cancer vaccine derived from telomerase-based helper peptides designed to stimulate a robust TH1 CD4 T cell response, used along with temozolomide (TMZ). Another promising candidate is berubicin, which works by intercalating into DNA strands to inhibit topoisomerase II activity and is administered following standard-of-care treatments. A recently completed phase III study also investigated enzastaurin hydrochloride, an inhibitor that targets protein kinase C and downstream signaling pathways, such as PI3K/AKT and MAPK. This study combined enzastaurin with radiotherapy and temozolomide (RT and TMZ) (NCT03776071). While conclusive results have yet to be published, interim findings on progression-free survival were not as promising when compared to outcomes seen with other therapeutic agents [202,204] (Table 2) (Figure 1).

## 5. Integrating Phytotherapy and Micotherapy in Glioblastoma Treatment

Phytotherapy and micotherapy are gaining attention as promising complementary approaches in the management of several pathophysiological conditions, from aging to cancer, e.g., in glioblastoma, colorectal, liver, prostatic, lung, and breast cancer treatment. By harnessing the anticancer properties of natural bioactive compounds, these therapies aim to boost the effectiveness of traditional treatments and offer new support in managing various cancer types [205,206,207,208,209,210,211,212,213]. These natural compounds exhibit various biological activities (anti-inflammatory, immunomodulatory, antioxidant, and antiproliferative effects) that may potentially improve treatment outcomes by targeting multiple cellular pathways associated with tumor growth, therapy resistance, and invasive properties. Notably, their selective cytotoxicity against GBM cells provides a safer therapeutic profile, making them ideal candidates for combination or adjuvant strategies in GBM therapy.

In a previous review, various phytotherapeutics were reported to benefit glioblastoma management, such as perrilyl alcohol, naringin, caffeine, artemisinin, and green tea extract, which consistently showed improved survival and reduced tumor volume with intranasal or oral administration of these compounds [214]. Recent in vitro studies on other natural compounds expand upon this knowledge.

Phytotherapy’s potential in GBM treatment is supported by studies highlighting the cytotoxic efficacy and selectivity of various plant-derived compounds. For example, the dichloromethane fraction from Mimosa caesalpiniifolia (Sabià) stem bark, rich in betulinic acid, which is known for its antioxidant and cytoprotective properties, shows effective targeting of GBM cells (SF-295), while sparing non-cancerous cells, by inducing cell cycle arrest [215]. Similarly, berberine, an alkaloid from Berberis vulgaris (Barberry), has shown promise by reducing U87MG GBM cell viability through G1-phase arrest and apoptosis. Berberine also enhances oxidative stress independently of conventional apoptosis pathways (AMPK, p53, and caspase-3), indicating its capacity to bypass traditional resistance mechanisms [216]. Another notable compound is quercetin, a flavonoid with antioxidant and anti-inflammatory effects, which modulates the tumor microenvironment by selectively reducing GBM cell viability and suppressing the Axl/IL-6/STAT3 signaling pathway, key elements in promoting tumor growth in vitro [217]. Phytochemicals like withanolides from Withania somnifera (Ashwagandha) and polyphenols from Castanea sativa (Chestnut) further underscore the role of natural compounds in modulating tumor-supportive signaling in GBM. Both compounds show in silico favorable binding to EIF4A3, a protein implicated in the regulation of oncogenic non-coding RNAs, suggesting a basis for their application in precision oncology to target specific molecular drivers of GBM progression [218].

Advances in delivery techniques for natural compounds are also enhancing their therapeutic efficacy against GBM. For example, α-mangostin from Garcinia mangostana (Mangosteen), when delivered via biotinylated and polysaccharide-modified PAMAM G3 dendrimers, showed increased solubility, selectivity, and anticancer activity against U-118 MG glioblastoma cells. Although GBM cells exhibited some resistance to this compound, likely due to limited mitochondrial targeting, the dendrimer conjugates reduced GBM cell adhesion and proliferation in vitro [219]. Another effective approach is using natural compounds in combination therapies. For instance, Rheum rhabarbarum (Rhubarb) extract paired with the oncolytic Newcastle disease virus produced a synergistic effect, enhancing immune responses and reducing tumor volume more effectively than monotherapies in vitro [220]. Similarly, resveratrol combined with 5-fluorouracil inhibited GBM cell proliferation in vitro by disrupting the Wnt/β-catenin signaling pathway and enhancing apoptosis through increased caspase-3 activation, which reduces the required doses of each compound, thereby lowering toxicity [221]. Muscone, a compound capable of crossing the blood–brain barrier, has also shown in vitro efficacy in overcoming TMZ resistance by targeting the FAK/EGFR/Integrin β1 pathway and inducing anoikis, a form of cell death, and DNA damage [222]. These combination therapies underscore the potential for phytotherapeutic agents to improve GBM sensitivity to standard chemotherapy and mitigate resistance. Also, curcumin, a bioactive compound in Curcuma longa (Turmeric), and polydatin, a resveratrol glucoside from Polygonum cuspidatum (Japanese knotweed), have shown promise in boosting TMZ effectiveness in in vitro models of GBM. As pretreatments, they reduced MGMT expression and disrupted autophagy in both MGMT-negative and -positive GBM cells [223]. Together, these studies support the use of natural compounds in GBM therapy as multi-target agents that complement conventional treatments, potentially improving outcomes by enhancing sensitivity, overcoming resistance, and engaging multiple therapeutic mechanisms.

In addition to phytotherapy, micotherapy offers a viable complementary approach to GBM treatment according to in vitro studies. The ethanolic extract from Trichoderma asperelloides has shown selective cytotoxicity against T98G glioblastoma cells, with minimal toxicity to non-cancerous cells. This selective action is particularly notable when compared to doxorubicin, as T. asperelloides demonstrated a similar level of efficacy but with a safer profile. This suggests it may serve as an effective standalone or adjuvant therapy to enhance chemotherapy efficacy while minimizing the risk to healthy tissues [224]. Another promising micotherapic agent is mycophenolic acid (MPA), a derivative from the Penicillium species, which is commonly used as an immunosuppressant but has shown anti-cancer effects by targeting inosine 5′-monophosphate dehydrogenase. MPA effectively downregulated TERT expression, a gene critical for tumor progression, and modulated MGMT levels, potentially enhancing chemotherapy response. MPA also exhibited synergy with BCNU, oxaliplatin, irinotecan, and TMZ, particularly in U251 GBM cells, where it increased apoptosis and reduced telomere length [225]. The role of medicinal mushrooms as adjuvants in GBM therapy alongside platinum-based chemotherapy was also demonstrated. Micotherapy impacted cell cycle progression, enhancing cell death signals and promoting apoptosis through mitochondrial pathways. Micotherapic supplements also influenced oxidative stress and led to necroptosis and ferroptosis, alternative cell death pathways, especially when combined with chemotherapy [209,226,227]. This indicates that micotherapy not only boosts chemotherapy’s effects but also activates distinct cell death mechanisms, potentially improving outcomes and counteracting glioblastoma resistance.

To address the limitations of natural compounds, such as poor bioavailability and challenges in crossing the blood–brain barrier, derivatives and advanced delivery systems have been developed. For instance, soloxolone para-methylanilide, a semisynthetic derivative of oleanolic acid, has shown enhanced efficacy against GBM by reducing invasiveness and promoting ROS-dependent apoptosis. When combined with TMZ, this derivative synergistically increased cytotoxicity in vitro and in U87 xenograft models [228]. Similarly, DIM (derivative of indole-3-carbinol) encapsulated in PLGA nanoparticles demonstrated improved BBB penetration and reduced toxicity. In combination with TMZ, these dual-loaded nanoparticles enhanced apoptosis markers, ROS production, and mitochondrial disruption, significantly reducing tumor growth in C6 xenograft models. This encapsulation approach has shown that natural compound derivatives can be optimized to target GBM’s complex biology, making them highly promising for future therapeutic strategies [229] (Table 3).

**Figure 2:**
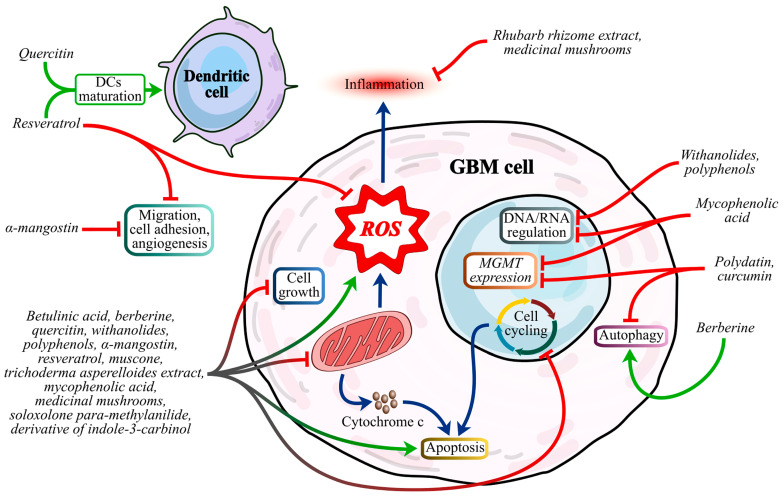
Schematic representation of phytotherapeutic and micotherapeutic strategies for glioblastoma. The diagram illustrates key molecular targets and pathways involved in GBM progression, highlighting therapies discussed in this review. Specifically, quercetin and resveratrol play a role in stimulating the maturation of dendritic cells. In particular, resveratrol also regulates oxidative stress in tumor cells while simultaneously inhibiting migration, cellular adhesion, and angiogenesis. These processes, i.e., vascular neogenesis, migration, and adhesion, are further negatively regulated by α-mangostin. Compounds such as betulinic acid, berberine, quercetin, withanolides, polyphenols, α-mangostin, resveratrol, muscone, trichoderma asperelloides extract, mycophenolic acid, medicinal mushrooms, soloxolone para-methylanilide, and a derivative of indole-3-carbinol demonstrate inhibitory effects on tumor growth, mitochondrial activity, and cell cycle. These compounds also impact oxidative stress pathways and stimulate apoptotic cell death mechanisms. Their mitochondrial action further affects the oxidative stress pathway, enhancing apoptotic signaling. Additionally, the release of mitochondrial cytochrome c and the influence on the cell cycle by these molecules drive the activation of apoptotic pathways. The regulation of DNA/RNA is inhibited by withanolides, polyphenols, and mycophenolic acid. Notably, mycophenolic acid also suppresses MGMT expression, an effect shared by polydatin and curcumin, both of which concurrently inhibit autophagy. However, autophagic cell death is promoted by berberine. Red arrows indicate inhibitory effects, while green arrows represent activation or promotion of therapeutic pathways. Abbreviations: DCs (dendritic cells), GBM (glioblastoma), MGMT (O6-methylguanine-DNA methyltransferase), ROS (reactive oxygen species).

Together, these studies underscore the potential of natural compounds in GBM therapy as multi-target agents that complement traditional treatments. By enhancing GBM sensitivity, overcoming resistance, and engaging multiple therapeutic mechanisms, phytotherapy and micotherapy could improve patient outcomes and reduce chemotherapy-related toxicity. Despite the fact that, to date, most studies on phytotherapy and micotherapy in glioblastoma have limited in vivo or clinical data, the multi-targeted actions of natural compounds in GBM remain highly promising. Addressing the need to test these compounds in complex settings to face challenges like blood–brain barrier penetration and bioavailability could be crucial to unlocking their potential as effective adjuvants in GBM therapy (Figure 2).

## 6. Conclusions and Future Directions in Glioblastoma Treatment

The inadequacy of gold-standard therapies for glioblastoma, combined with the challenges posed by the tumor itself, often results in treatment failures. This reality highlights the urgent need for multi-targeted therapeutic strategies. Current treatment options have significant limitations, leading to disappointing outcomes in numerous clinical trials. The complexity of GBM, characterized by its resistance mechanisms and tumor heterogeneity, calls for a shift toward more integrated treatment approaches.

To address these challenges, researchers are increasingly evaluating combination therapies that exploit synergistic effects to enhance treatment efficacy. Ongoing clinical trials are exploring various combinations of agents to improve patient outcomes. This shift underscores the importance of integrating both conventional therapies and innovative approaches, potentially leading to more effective treatment regimens.

In this context, phytotherapy and micotherapy offer promising avenues for complementing traditional GBM treatments. These therapies utilize natural bioactive compounds known for their anti-inflammatory, immunomodulatory, and antiproliferative properties. Various plant-derived compounds have shown selective cytotoxicity against GBM cells while sparing healthy cells, which could provide a safer therapeutic profile. Moreover, incorporating these natural compounds may enhance the efficacy of standard treatments. While specific clinical trials assessing phytotherapy and micotherapy in GBM are still limited, ongoing studies investigating the effects of these natural compounds on immune modulation and tumor growth inhibition are noteworthy. Integrating phytotherapy and micotherapy into GBM treatment strategies could lead to innovative regimens that enhance efficacy, reduce side effects, and improve patients’ QoL. To effectively integrate these approaches, it will be crucial to validate these strategies and elucidate their mechanisms of action within the context of GBM. Additionally, establishing standardized dosages and administration routes will be essential for successful implementation. Continued exploration of these natural compounds in clinical settings is vital for confirming their roles in GBM management.

## Figures and Tables

**Table 1:** Therapeutic strategies and experimental treatments for glioblastoma. This table provides an overview of therapeutic strategies under investigation for glioblastoma, providing details on the type of therapy, therapeutic class, underlying mechanism, monotherapy effectiveness, and notable limitations or challenges associated with each strategy. Abbreviations: ACNU (nimustine), AKT (protein kinase B), BBB (blood–brain barrier), BCNU (carmustine), CAR-NK/T (chimeric antigen receptor natural killer/T cells), CCNU (lomustine), CED (convection-enhanced delivery), CNS (central nervous system), DNA (deoxyribonucleic acid), EGFR (epidermal growth factor receptor), GBM (glioblastoma), GLS (glutaminase), HDAC (histone deacetylase), HSV (herpes simplex virus), IDH (isocitrate dehydrogenase), IGV-001 (immunotherapy inducing immunogenic cell death), LDH (lactate dehydrogenase), mRNA (messenger ribonucleic acid), mTOR (mechanistic target of rapamycin), PARP (poly(ADP-ribose) polymerase), PD-1 (programmed death 1), PDGFR (platelet-derived growth factor receptor), PI3K (phosphatidylInositol 3-kinase), RTK (receptor tyrosine kinase), TME (tumor microenvironment), TMZ (temozolomide), VEGF (vascular endothelial growth factor), VEGFR (vascular endothelial growth factor receptor.

Therapy	Therapeutic Class	Mechanism	Monotherapy Effectiveness	Limitations and Notes
**ACNU [42,43,44]**	Chemical compound, nitrosoureas	Alkylation (DNA cross-linking), protein carbonylation	Effective in vitro and in vivo in TMZ-resistant cells, especially via CED	High toxicity in preclinical models
**BCNU and CCNU [42,45,46,47,48,49,50,51,52,53]**	Chemical compound, nitrosoureas	Alkylation (DNA cross-linking), protein carbonylation	Comparable to TMZ, no clinical relevance	High toxicity in preclinical models
**Cisplatin, Carboplatin, and Derivatives [54,55,56,57,58]**	Chemical compound, platinum derivatives	Alkylation (DNA cross-linking)	Effective when locally delivered according to clinical studies	Systemic toxicity and poor BBB penetration in preclinical and clinical settings
**CAR-T and CAR-NK Cell Therapy [59,60,61,62,63,64,65]**	Genetically engineered cells, immunotherapy	Genetically engineered cells target GBM surface antigens	Promising in vitro and preclinical studies; no clear survival benefit in clinical trials	Immunosuppressive TME in in vitro and preclinical studies; NK cells have fewer toxicities than T cells
**Pembrolizumab [65,66,67,68]**	Monoclonal antibody, immune checkpoint inhibitor	PD-1 inhibitor	No significant survival benefit in clinical settings	Limited efficacy in GBM due to immunosuppressive tumor environment
**Nivolumab [69,70,71,72]**	Monoclonal antibody, immune checkpoint inhibitor	PD-1 inhibitor	Promising in rodent models; moderate immune response in patients, but limited effectiveness	Variable patient response; limited BBB penetration
**IGV-001 [73,74,75]**	Peptide-based immunotherapy	Inductor of immunogenic cell death	Promising preclinical results; undergoing clinical trials for newly diagnosed GBM	No notes to date
**Cancer Vaccines (mRNA [76,77,78,79], DNA [80,81,82,83], Dendritic Cell [84,85,86,87,88])**	Viral vector or cell-based vaccine	Immune stimulation	Preliminary promising according to in silico analysis and in preclinical studies, but limited improvement in survival in clinical settings	Contributed to immune suppression in TME; adverse CNS effects emerged in clinical studies
**Oncolytic Virus (HSV, Flavivirus, Adenovirus) [89,90,91,92,93,94,95,96,97,98]**	Viral therapy	Selectively replicates in and kills tumor cells	Promising in vitro and mouse models; different response in patients	Insufficient delivery to tumor; increased expression of immune checkpoints
**RTK Inhibitors [104,105,106,107,108,109,110,111,112,113]**	Small molecule, tyrosine kinase inhibitors	Inhibits receptor tyrosine kinases like EGFR and PDGFR	Therapeutic interest observed from in silico to preclinical studies. Limited efficacy and mixed outcomes have been obtained in clinical settings (where such an advanced stage was achieved)	Tumor heterogeneity and resistance mechanisms emerged in preclinical and clinical settings
**VEGF Inhibitors (Bevacizumab) [114,115,116,117,118,119,120]**	Monoclonal antibody, anti-angiogenic agent	Inhibits angiogenesis	Modest impact on survival in preclinical and clinical studies	Compensatory pathways and GBM molecular heterogeneity
**PI3K/AKT/mTOR Pathway Inhibitors (Buparlisib, Everolimus, Ipatasertib) [126,127,128,129,130,131,132,133]**	Small molecules, pathway inhibitors	Targets PI3K, AKT, and mTOR for cell growth regulation	Limited efficacy and mixed outcomes in vitro and in vivo	Compensatory pathways and GBM molecular heterogeneity resulted in variable therapeutic response in clinical studies
**Autophagy and Proteasome Inhibitors (Marizomib) [134,135,136]**	Small molecule, proteasome inhibitor	Inhibits proteasome to disrupt cellular metabolism	Limited benefit in clinical studies	Exacerbates chemotherapy side effects
**Metabolic Targeting (IDH, LDH, GLS Inhibitors) [137,138,139,140,141,142,143,144,145,146,147]**	Small molecules, metabolic modulators	Alters cellular metabolism by inhibiting metabolic enzymes	Promising in silico and in some preclinical models	Compensatory metabolic pathways activation observed in some preclinical studies
**Starvation-Based Metabolism Modifiers [121,148,149,150,151]**	Nutritional intervention	Glucose/arginine deprivation, ketogenic diet to limit tumor growth	Promising results in preclinical and clinical studies for glucose starvation	Ketogenic diet may have immunosuppressive effects on macrophages according to preclinical evaluation
**HDAC Inhibitors (Panobinostat, Valproic Acid) [152,153,154,155,156,157,158,159,160]**	Small molecules, epigenetic modulators	Modulates epigenetic gene expression and chromatin organization	Promising effects in vitro and in preclinical studies but no noteworthy benefits in clinical trials	Systemic toxicity, poor BBB penetration and GBM molecular heterogeneity
**PARP Inhibitors (Niraparib, Olaparib) [161,162,163,164,165,166,167,168,169]**	Small molecules, DNA repair inhibitors	Inhibits DNA repair mechanisms	Promising effects in preclinical trial but irrelevant efficacy in preclinical trials as monotherapy	Compensatory pathways and GBM molecular heterogeneity
**Metformin [170,171,172,173]**	Small molecule, metabolic modulator	Gluconeogenesis inhibitor; enhances glucose metabolism	Effectiveness showed in vivo and clinical studies	Systemic toxicity and poor BBB penetration are limiting factors
**Disulfiram [174,175,176]**	Small molecule, metabolic modulator	Aldehyde dehydrogenase inhibitor	Shows potential in disrupting GBM metabolism in preclinical and clinical settings	Systemic toxicity and poor BBB penetration are limiting factors
**Chloroquine [157,177,178,179]**]	Small molecule, autophagy modulator	Modulates autophagy and cell metabolism	Modulates cancer metabolism in vitro and is still under investigation; some clinical benefit in combination therapies	Systemic toxicity and poor BBB penetration are limiting factors
**Proton Therapy [180,181,182]**	Radiation therapy	Uses Bragg peak to target tumors precisely	Promising as adjuvant in clinical studies, but long-term effectiveness uncertain	Limited by delivery depth and tumor heterogeneity, as monotherapy in preclinical and clinical trials the efficacy is not certain to date
**Sonodynamic/Photodynamic Therapy [183,184,185,186,187,188,189,190]**	Non-invasive therapy	Uses ultrasound/light waves to activate sensitizers within tumor	Promising ROS production; under investigation from in vitro to in clinic	Depth of the tumor, heterogeneity, and hypoxia impact efficacy
**Hyperthermia Therapy [191,192,193,194]**	Adjunctive thermal therapy	Increases tumor temperature to sensitize cells to chemotherapy	Ineffective alone; promising in combination therapies	Complex tumor vascularization limits effectiveness according to in silico studies
**Tumor Treating Fields [195,196,197,198,199,200,201]**	Physical therapy	Alternating electric fields disrupt cancer cell division	Effective according to clinical trials but with heterogenous response among patients	Compliance challenges and variable patient response

**Table 2:** Combination therapies under investigation in clinical trials for glioblastoma treatment according to the NIH website. The table summarizes various therapeutic strategies and combination trials for glioblastoma, categorized by the therapeutic agents, trial details, clinical phase, objectives and known effects. Abbreviations: AB154 (anti-TIGIT monoclonal antibody), AB122 (zimberelimab, anti-PD-1 monoclonal antibody), AGuIX (advanced gadolinium-based nanoparticles), BET (bromodomain and extra-terminal domain protein family), CDK4/6 (cyclin-dependent kinase 4/6), CTLA-4 (cytotoxic T-lymphocyte associated protein 4), DDR (DNA damage repair), ERK1/2 (extracellular signal-regulated kinase 1/2), GBM (glioblastoma), PD-1 (programmed death 1), PDE5 (phosphodiesterase 5), PKC (protein kinase C), RAF/MEK (rapidly accelerated fibrosarcoma/mitogen-activated protein kinase kinase), RT (radiotherapy), SurVaxM (Vaccine targeting Survivin), TIGIT (T cell immunoreceptor with immunoglobulin and ITIM domains), TME (tumor microenvironment), TMZ (temozolomide), UCPVax (universal cancer peptide vaccine), VEGFR (vascular endothelial growth factor receptor).

Therapeutic Agents	Combination Therapy/Trial Details	Clinical Phase	Objective/Known Effects
**Tinostamustine** **+ TMZ + Radiation**	Dual-action alkylating agent + standard therapy	Phase I	Targets both cancer cells and TME; aims to increase sensitivity to radiotherapy and delay recurrence
**AZD1390, Niraparib, Pamiparib, Olaparib** **+ Radiation**	DDR (DNA damage repair) inhibitors + radiation	Phase I	Expected to improve radiation efficacy by blocking DNA repair in tumor cells
**Pamiparib (BGB-290)** **+ TMZ**	DDR inhibitor + TMZ chemotherapy	Phase I/II	Aims to exploit DNA repair deficiencies in GBM cells to enhance TMZ efficacy
**LY3214996** **+ Abemaciclib**	ERK1/2 inhibitor + CDK4/6 inhibitor	Preclinical	Expected to synergize in controlling cell cycle and inhibiting tumor growth
**AB154 (Anti-TIGIT)** **+ AB122 (Zimberelimab, Anti-PD-1)**	Dual checkpoint inhibition	Phase I	Aims to boost immune response against GBM cells, potentially overcoming immune suppression within TME
**Defactinib + VS-6766**	Focal adhesion kinase inhibitor + RAF/MEK inhibitor	Preclinical	Intended to inhibit pathways involved in cell adhesion and proliferation
**Personalized** **High-Throughput Screened Drug** **Cocktail**	Patient-derived cancer stem cell-targeted drugs + standard therapy	Phase I	Individualized combination aiming to enhance efficacy based on specific tumor profile; effectiveness varies by patient
**Chlorpromazine,** **Cannabinoids,** **Chloroquine** **+ Radiation + TMZ**	Various agents with anti-inflammatory and antipsychotic properties + standard therapy	Phase I	Potential anti-angiogenic, autophagy-modulating effects; seeking to improve tumor response to standard therapy
**Tadalafil** **(PDE5 inhibitor),** **CC-90010 (BET inhibitor) + Radiation + TMZ**	Vasodilatation factor + transcription factor inhibitor + standard therapy	Phase I	Targeting specific pathways involved in tumor growth
**AGuIX Nanoparticles + Radiation + TMZ**	Radiosensitizer nanoparticles + standard therapy	Phase I/II	Expected to enhance radiation delivery and tumor targeting, increasing tumor response to radiation
**Dendritic Cell Vaccine + Radiation + TMZ**	Personalized immune stimulation + standard therapy	Phase I/II	Seeks to generate strong immune response and tumor antigen recognition; early studies show potential for prolonging survival
**Nivolumab,** **Pembrolizumab,** **Cemiplimab,** **Spartalizumab** **+ CTLA-4 Inhibitors**	PD-1 inhibitors + CTLA-4 inhibitors	Phase I-III	Aims to break immune suppression in TME and enable more effective immune attack on GBM cells
**Nivolumab** **+ Regorafenib (VEGFR inhibitor)**	Immune evasion inhibitor + angiogenesis inhibitor	Phase II	Recently completed; efficacy results do not support further evaluation in GBM due to limited benefit
**Pembrolizumab** **+ Lerapolturev** **(Oncolytic Virus)**	PD-1 inhibition combined + oncolytic virus	Phase II	Expected to enhance immune response through direct oncolysis and immune activation
**Azeliragon + Radiation**	Anti-inflammatory + radiation	Phase II	Intended to reduce inflammation, potentially improving radiation response
**UCPVax** **(Telomerase-derived vaccine) + TMZ**	Anti-cancer vaccine targeting telomerase + TMZ	Phase II	Targeting telomerase in GBM cells to enhance immune response; early results show potential for improving survival
**Berubicin** **(Topoisomerase II** **inhibitor) + Standard of Care**	Topoisomerase II + standard therapy	Phase II	Targets DNA replication, potentially effective in aggressive tumors
**Enzastaurin** **Hydrochloride + RT** **+ TMZ**	PKC pathway inhibitor + standard therapy	Phase III	Interim results suggest limited impact on progression-free survival compared to other treatments; further data needed
**EO2401/Imiquimod** **+ Nivolumab/Bevacizumab**	Immunomodulatory agents + VEGFR inhibitor	Phase II	Enhances immune cell recognition and infiltration; early studies show promise for increasing progression-free survival
**SurVaxM (Peptide vaccine) + Pembrolizumab**	Vaccine targeting survivin + PD-1 inhibitor	Phase II	Aims to increase survival by targeting survivin-expressing tumor cells
**Phytotherapy and Micotherapy with** **Traditional Therapies**	Natural compounds with anti-tumor properties + hemotherapies	Preclinical	Expected to enhance therapeutic response by targeting tumor growth, therapy resistance, and immunomodulation with lower toxicity; selective GBM cell cytotoxicity offers potential for complementary or adjuvant strategies in GBM.

**Table 3:** Natural compounds and extracts investigated for glioblastoma therapy. This table includes natural compounds sources, study types, therapeutic mechanisms, and observed effects or outcomes in preclinical studies. Abbreviations: 5-FU (5-fluorouracil), BBB (blood–brain barrier), BCNU (carmustine), DOX (doxorubicin), EGFR (epidermal growth factor receptor), FAK (focal adhesion kinase), GBM (glioblastoma), IL-6 (interleukin-6), MGMT (O6-methylguanine-DNA methyltransferase), PLGA (poly(lactic-co-glycolic acid), ROS (reactive oxygen species), STAT3 (signal transducer and activator of transcription 3), TERT (telomerase reverse transcriptase), TMZ (temozolomide).

Natural Compound/Extract	Source	Study Type	Therapeutic Mechanisms	Effects/Outcomes
**Betulinic acid** (Dichloromethane fraction) [215]	*Mimosa caesalpiniifolia*	In vitro (SF-295 cells)	Antioxidant, cytoprotective; induces cell cycle arrest	Selective cytotoxicity against GBM cells; spares non-cancerous cells by targeting cell cycle in cancer cells
**Berberine [216]**	*Berberis vulgaris*	In vitro (U87MG cells)	G1-phase arrest, apoptosis enhancement, oxidative stress induction independent of apoptosis pathways	Reduces GBM cell viability; bypasses conventional apoptosis resistance mechanisms
**Quercetin [217]**	Various plants	In vitro (GBM cells)	Modulates tumor microenvironment; targets Axl/IL-6/STAT3 pathway	Reduces GBM cell viability and suppresses signaling pathways that promote tumor growth
**Withanolides [218]**	*Withania somnifera*	In silico models and computational predictions	Targets EIF4A3, involved in oncogenic RNA regulation	Inhibits GBM cell growth by disrupting non-coding RNA pathways implicated in tumor progression
**Polyphenols [218]**	*Castanea sativa*	In silico models and computational predictions	Binds EIF4A3; modulates signaling	May suppress GBM-promoting non-coding RNAs, providing specificity for GBM cells
**α-Mangostin** (via dendrimer delivery) [219]	*Garcinia mangostana*	In vitro (U118 MG cells)	Increases solubility and selectivity; reduces cell adhesion and proliferation	Enhanced targeting of GBM cells with reduced off-target effects; limited mitochondrial targeting may impact efficacy
**Rhubarb Rhizome** **Extract + Newcastle Disease Virus [220]**	*Rheum rhabarbarum*	In vitro (AMGM5 cells)	Immune response enhancement, oncolytic virus synergy	Synergistic effect increases immune response and reduces tumor volume
**Resveratrol** **+ 5-Fluorouracil [221]**	Various plants	In vitro (U87 cells)	Disrupts Wnt/β-catenin pathway, increases caspase-3 activity	Inhibits GBM cell proliferation; requires lower compound doses, reducing toxicity
**Muscone [222]**	*Moschus moschiferus*	In vitro (U251 cells)	Induces anoikis and DNA damage, targets FAK/EGFR/Integrin β1 pathway	Effective in overcoming TMZ resistance, promotes cell death specific to GBM cells
**Curcumin** **and Polydatin [223]**	*Curcuma longa*, *Polygonum cuspidatum*	In vitro (U87 and LN18 cells)	Lowers MGMT expression, disrupts autophagy	Enhances TMZ effectiveness in both MGMT-negative and -positive GBM cells
**Trichoderma** **asperelloides Extract [224]**	*Trichoderma asperelloides*	In vitro (T98G cells)	Reduce tumor cells viability at low doses, sparing healthy cells	Similar efficacy to doxorubicin, with reduced side effects; potential as an adjuvant therapy with DOX and 5-FU
**Mycophenolic Acid (MPA) [225]**	*Penicillium* species	In vitro (U251 cells)	Downregulates TERT, modulates MGMT, apoptosis enhancement	Synergizes with BCNU, oxaliplatin, irinotecan, and TMZ; reduces telomere length and increases chemotherapy sensitivity
**Medicinal Mushrooms + Platinum-based Chemotherapy [209,226,227]**	Various mushrooms	In vitro (U251 cells)	Promotes oxidative stress, induces necroptosis and ferroptosis	Enhances chemotherapy response and activates multiple cell death pathways
**Soloxolone** **para-methylanilide (Oleanolic acid** **derivative) [228]**	*Olea europaea*	In vitro and in vivo (U87 xenografts)	ROS-dependent apoptosis, reduces invasiveness	Enhances cytotoxicity when combined with TMZ; reduces tumor invasiveness and growth in animal models
**DIM (Deriv. of** **indole-3-carbinol)** (PLGA nanoparticles) [229]	Various cruciferous vegetables	In vivo (C6 xenografts)	Improved BBB penetration, enhances apoptosis and ROS production	Reduced tumor growth in GBM animal models; potential for enhanced delivery and efficacy

## Data Availability

No new data were created or analyzed in this study. Data sharing is not applicable to this article.
